# Two Randomized Trials Provide No Consistent Evidence for Nonmusical Cognitive Benefits of Brief Preschool Music Enrichment

**DOI:** 10.1371/journal.pone.0082007

**Published:** 2013-12-11

**Authors:** Samuel A. Mehr, Adena Schachner, Rachel C. Katz, Elizabeth S. Spelke

**Affiliations:** 1 Department of Psychology, Harvard University, Cambridge, Massachusetts, United States of America; 2 Harvard Graduate School of Education, Cambridge, Massachusetts, United States of America; University of Tuebingen Medical School, Germany

## Abstract

Young children regularly engage in musical activities, but the effects of early music education on children's cognitive development are unknown. While some studies have found associations between musical training in childhood and later nonmusical cognitive outcomes, few randomized controlled trials (RCTs) have been employed to assess causal effects of music lessons on child cognition and no clear pattern of results has emerged. We conducted two RCTs with preschool children investigating the cognitive effects of a brief series of music classes, as compared to a similar but non-musical form of arts instruction (visual arts classes, Experiment 1) or to a no-treatment control (Experiment 2). Consistent with typical preschool arts enrichment programs, parents attended classes with their children, participating in a variety of developmentally appropriate arts activities. After six weeks of class, we assessed children's skills in four distinct cognitive areas in which older arts-trained students have been reported to excel: spatial-navigational reasoning, visual form analysis, numerical discrimination, and receptive vocabulary. We initially found that children from the music class showed greater spatial-navigational ability than did children from the visual arts class, while children from the visual arts class showed greater visual form analysis ability than children from the music class (Experiment 1). However, a partial replication attempt comparing music training to a no-treatment control failed to confirm these findings (Experiment 2), and the combined results of the two experiments were negative: overall, children provided with music classes performed no better than those with visual arts or no classes on any assessment. Our findings underscore the need for replication in RCTs, and suggest caution in interpreting the positive findings from past studies of cognitive effects of music instruction.

## Introduction

Young children's lives are saturated with musical activities: parents worldwide sing regularly with their children and most preschool programs incorporate musical activities into their curricula (for review, see [1]). In spite of the pervasiveness of preschool music activities, however, the effects of early music education on children's cognitive development remain unclear. Many studies have reported associations between music training and improvements in cognitive skills [e.g., 2–3], though the largest correlational study on the topic reported no such effect [4]. Only five published randomized controlled trials (RCTs) have investigated causal effects of music training on areas of cognition seemingly unrelated to music [5]–[9] – effects that would support the often-repeated claim that “music makes you smarter” – and no clear pattern of results has emerged.

Schellenberg [7] reported a significantly greater increase in general intelligence (as measured by the Wechsler Intelligence Scale for Children—Third Edition; WISC-III [10]) in children randomly assigned to keyboard or voice lessons, compared to those taking drama or no lessons. However, two subsequent RCTs failed to find corresponding IQ effects with other types of music training [8]–[9]. Moreno et al. [8] administered the WISC-III after Kodàly music or painting training and found no evidence for a greater increase in IQ in the music group. In a second study, Moreno et al. [9] administered two subtests of the Wechsler Preschool and Primary Scale of Intelligence—Third Edition (WPPSI-III [11]), an IQ test for younger children that corresponds to the WISC-III, after computer-based music or visual arts training. The music group significantly outperformed the visual arts group on the Vocabulary subtest but not the Block Design subtest; the authors reported no overall effect of music training on general intelligence [9]. Two further RCTs reported similarly mixed results: Costa-Giomi [5] found significant increases in children's general intelligence (as measured by the Developing Cognitive Abilities Test [12]) after two years of piano lessons, but not after one or three years. Bilhartz, Bruhn, and Olson [6] tested kindergarteners on subtests of the Stanford-Binet Intelligence Scale [13] after group parent-child music classes and found significant increases in performance on one subtest, but no overall effect of music training on general intelligence. Thus, the current literature does not provide a definitive answer regarding the nonmusical cognitive effects of music training.

These five RCTs vary widely in terms of the age of children tested, the type of music training provided, and the outcome measures used (see Table 1). Thus, there are two possible explanations for such inconsistent findings: either different music training methods produce different cognitive effects and the various findings accurately identify what training content is necessary to induce each type of effect, or some proportion of the studies report false positives or false negatives.

**Table 1:** Review of previous RCTs assessing child cognitive development and music lessons.

Notes on Main or Ancillary Effects	Music group outperforms control group on overall test after 2 years of training in a direct comparison of scores (Tukey test, *p* = .05), but not after 1 or 3 years.	Music group shows significantly larger increase in performance than control group on 1 of 5 subtests (Bead Memory; *F*(1,43) = 6.29, *p* = .016).	Combined keyboard and voice group shows significantly larger increase in *g* than combined drama and control group (*t*(130) = 1.99, *p* = .049, *d* = .184^3^).	Music group has significantly fewer errors on 1 of 3 subsets of the reading battery (Tukey test, *p<*.05^2^)	Music group shows significant increase in performance on 1 of 2 subtests (Vocabulary; *F*(1,62) = 11.37, *p* = .0013, partial *η* 2 = .33), visual art group does not.
**Citation Count** 1	167	124	374	183	59
**Correction for Multiple Comparisons?**	Yes	No	No	No	No
**Overall Cognitive Effect?**	No	No	Yes	No	No
**Additional Measures Administered**	Musical Aptitude Profile [49]; Bruininks-Oseretsky Test of Motor Proficiency [50]; Canadian Achievement Test 2 [51]; Coopersmith Self-Esteem Inventories [52]	Young Child Music Skills Assessment (designed by authors)	Kaufman Test of Education Achievement [54]; Behavioral Assessment System for Children [55]	Portuguese European Reading Battery [59]; tests of speech and pitch discrimination [60] with EEG recording	Executive function “go/no-go” task (designed by authors) with EEG recording
**Primary Measure of Child Cognition**	*g* via Developing Cognitive Abilities Test ([12], 3 of 3 subtests administered)	*g* via Stanford-Binet Intelligence Scale ([13], 5 of 15 subtests administered)	*g* via Wechsler Intelligence Scale for Children ([10], 12 of 12 subtests administered)	*g* via Wechsler Intelligence Scale for Children, Portuguese adaptation ([58], 10 of 10 subtests administered)	*g* via Wechsler Preschool and Primary Scale of Intelligence ([13], 2 of 7 subtests administered)
**Training Length**	52.5 hours over 90 weeks	37.5 hours over 30 weeks	28 hours over 36 weeks	55 hours over 24 weeks	15 hours over 4 weeks
**Comparison Group Type**	No-treatment control	No-treatment control	Weekly group drama lessons or no-treatment control	Weekly group painting lessons	Daily computer-based group visual art activities
**Music Curriculum**	Weekly private piano lessons with “traditional curriculum”	Weekly Kindermusik [38] group classes (sometimes includes parents)	Weekly group “standard” piano lessons or Kodály [53] voice lessons	Weekly group classroom music in Kodály, Orff, and Wuytack methods [57]	Daily computer-based group music listening activities
**Age of Children at Testing (in years)**	12^2^	4.9–6.7^2^	7.08 (.237)	8.82 (.375)	5.40 (5.65)
**Final Sample Size**	67	66	132	32	48
**Initial Sample Size**	117	71	144	37	64
**Study**	[5]	[6]	[7]	[8]	[9]

*Note*. Studies are reviewed in chronological order, from 1999 [5] to 2011 [9]. Standard deviations, where available, are in parentheses. This table was completed with the generous assistance of the authors of each of the above papers, who helped clarify a variety of relevant statistics from their work.

1 As of November 2013, from Google Scholar.

2 Value represents an estimate; exact value was not available from the author(s).

3 The effect size reported in [7] is *d* = .35, a figure calculated in terms of the pooled standard deviation of children's increases in IQ score. However, the relevant effect size is in terms of the standard deviation of the test itself, not that of gain scores (see, e.g., [56]). Thus, we recalculated the effect size using the standard deviation of the WISC-III's reference population (fifteen IQ points [10]) and report this figure above.

Two considerations favor the latter explanation. First, previous RCTs present results without correcting for multiple comparisons; in some cases, after such a correction the results are statistically nonsignificant (e.g., [6]; for discussion, see [14]). Second, the existing literature lacks published replications. In each of the five articles discussed above, only one RCT was conducted and to date, no relevant RCT has directly replicated any of the above findings (i.e., using the same music intervention and outcome measures). The lack of published replications makes this literature vulnerable to publication bias, because positive findings are more likely to be published than null findings. To resolve possible publication bias and allow for accurate meta-analysis of the literature, it is necessary to publicize null results and strict replication attempts in addition to novel positive findings [14]–[15].

In the current study, we investigated the effects of parent-child music education on specific cognitive skills in preschool children, who were compared to children of equally motivated parents who either received a different form of arts instruction (parent-child visual arts education, Experiment 1) or who received the same music instruction after rather than before the cognitive testing (no-treatment control group, Experiment 2). Instead of IQ subtests, we used measures of distinct areas of cognitive development in which music- and arts-trained students have been reported to excel: spatial-navigational reasoning, visual form analysis, numerical discrimination, and receptive vocabulary.

There are several reasons why measures of specific areas of cognition may be more informative than previously used tests of general intelligence. First, IQ subtests each tend to be brief, so as to maintain the child's attention over many assessments (the WISC-III has 12 individual subtests [10]). As a result, less information is collected for each individual skill, in comparison to an in-depth cognitive test focusing on that skill alone. For instance, the WPPSI-III Vocabulary subtest assesses children on the meanings of just 25 words [11], while the Peabody Picture Vocabulary Test (PPVT; [16]), a standardized test of receptive vocabulary, typically assesses children on two to three times that number of words (test length and item difficulty are adapted to the child's skill level, with a maximum of 204 items [16]). This difference likely contributes to the PPVT's substantially higher test-retest reliability (*r* = .95 for ages 4.5–4.9 [16]) relative to the WPPSI-III Vocabulary subtest (*r* = .68 for ages 4.0–5.4 [11]). Furthermore, IQ subtests are single components of a factor analysis, and thus are not designed as standalone measures [17]. Because of their poor reliability [18] and questionable external validity [19], researchers have cautioned against using IQ subtests as stand-alone measures to describe specific cognitive skills [20] or as aggregate measures of group performance [21].

Thus, in the current study we chose to use tasks designed to measure performance within specific cognitive domains that hold promise for revealing cognitive effects of music training. Numerous correlational studies have reported associations between musical and mathematical or verbal abilities (for review, see [22]). Moreover, recent research shows that individual differences in sensitivity to numerical differences in simple, non-symbolic arrays of dots correlate with individual differences in mathematical ability in the preschool years [23], as well as at a wide range of older ages, across cultures [24]–[27].

A correlational study of older students also provides evidence for an association between training in music or visual arts and two of the spatial-cognitive abilities that we measure: in an intensive arts high school, music and dance students showed greater ability to use geometric maps to navigate 3-D space than visual arts students, but were no better than visual arts students at analyzing the geometric properties of 2-D visual forms. Conversely, the duration of visual arts training significantly predicted ability to use geometric properties to analyze 2-D visual forms, whereas music training did not predict this ability [28]. These two tests of spatial ability can be administered over a wide range of ages, from preschoolers to adults, and show similar performance patterns across age groups [29]. Thus, tests of map-based navigation, visual form analysis, numerical discrimination, and receptive vocabulary may be more sensitive measures of effects of music instruction on young children's cognitive abilities than the IQ tests employed in previous RCTs.

The current study also adds to existing literature by studying effects of *parent-child* music instruction on preschool children's cognitive development, as opposed to individual music lessons, which children typically attend without a parent. This type of instruction is a prevalent form of early music exposure, endorsed by educators in the United States [1], [30], and yet has rarely been studied. It may hold particular promise for revealing effects of music training, because training programs that include parents may alter their behavior at home, amplifying the effects of the music training. Relatively brief, play-centered music interventions are also less likely to introduce selective attrition by children who are less able to focus and persevere on structured tasks: qualities that are related to later cognitive skills [31] and academic achievement [32]. We focus on this type of music instruction both for these reasons, and because this focus allows us to probe the cognitive effects of typical preschool children's music enrichment activities. In each experiment, we employ randomized subject assignment across treatment groups, while equating groups on demographic factors, selected cognitive characteristics, and parental music aptitude. We also use the same teacher for all classes, so as to help control for teacher characteristics, and to ensure that parents and children have comparable relationships with the teacher across the two class types. Data were collected soon after the training ended, by experimenters who were unaware of participants' assigned training condition and who had no previous contact with the children or parents.

## Experiment 1

We randomly assigned 29 four-year-old children to music or visual arts classes, controlling for a variety of characteristics (e.g., age, receptive vocabulary, family income). Parents accompanied their children to six weekly 45-minute classes. The curricula were designed to foster parent-child play in the context of arts media (music or visual arts). For instance, parents sang lullabies to their children in the music class, and worked on crafts projects with their children in the visual arts class. Before subject assignment, we tested children on receptive vocabulary and collected demographic data. After six weeks of class, the children returned to our lab for a single posttest session where a team of investigators assessed their performance in two domains of spatial reasoning (map-based navigation and visual form analysis), numerical discrimination, and receptive vocabulary.

### Methods

#### Ethics Statement

Study protocols were approved by Harvard University's Institutional Review Board, the Committee on the Use of Human Subjects in Research. Informed consent was obtained in writing from the parents/guardians on behalf of the child participants and verbal consent was obtained from the children. Either the guardians or children could end their participation at any time.

#### Participants

We recruited families with four-year-old children through a lab database and by distributing flyers offering “Free Creative Arts Classes” throughout the Boston area. Approximately 40 families responded, of which 32 were invited to participate in the full study on the basis detailed below. One child from each group (music or visual arts) discontinued participation after the first day of classes and one additional child from the visual arts group failed to attend any sessions, for a 9.4% rate of attrition (after attrition: music group: *n* = 15, 7 female; visual arts group: *n* = 14, 6 female).

#### Pretest and Subject Assignment

Figure S1 describes the chronology of participation in Experiment 1. Participants took part in pretest assessments during individual sessions (Nov. 13–Dec. 13, 2010). Form A of the PPVT-III [16] was administered to children and the Advanced Measures of Music Audiation, a standardized measure of adult music aptitude, was administered to parents (AMMA; [33]). If a child was accompanied by both parents at pretest, the parent who planned to accompany the child to class took the AMMA. Parents also answered written questions on family income and ethnicity, the child's current participation in arts classes, and the presence of any professional artists currently living in the child's home. Eight children were excluded because they were currently attending a music class, a professional musician was currently living at home with the child, or the parent reported that they were unavailable for one or more classes.

Participants were randomly assigned to the music or visual arts groups via a MATLAB script that generated 80,000 possible groupings and returned the grouping with the smallest differences between groups in terms of age, gender distribution, family income, ethnicity, child PPVT-IIIa score, and parent AMMA score (see Table 2).

**Table 2:** Mean characteristics and pretest performance in Experiments 1 and 2.

	Experiment 1		Experiment 2		
Characteristic	Music	Visual Arts	Music	Control	
Age at posttest (years)	4.86 (.307)	4.64 (.268)	4.71 (.260)	4.72 (.353)	
Family income (thousands of dollars per year)	136 (74.8)	116 (48.0)	155 (64.5)	135 (53.4)	
Both parents' total work hours per week	66.14 (24.1)	75.1 (16.9)	67.8 (19.9)	67.7 (19.4)	
Parent's AMMA score (% correct)	65.3 (6.36)	69.4 (11.2)	67.9 (9.05)	66.1 (7.52)	
Child's PPVT-IIIa score (age-standardized)	117 (13.6)	117 (14.6)	119 (9.46)	120 (10.1)	

*Note*. Standard deviations are in parentheses. Because of the rapid rate of vocabulary acquisition in preschool children, PPVT scores are standardized by age to enable direct comparison from pre- to posttest.

#### Training

Four classes (two music, two visual arts) were conducted, with seven to eight participants per class (four male and three or four female). All classes met in the same room on Saturday mornings, with 45-minute sessions weekly for six weeks (Jan. 8–Feb. 12, 2011). Attendance was high in both the music (92.2%; 7 absences and 83 attendances) and visual arts groups (84.5%; 13 absences and 71 attendances), with no significant difference between groups (χ^2^(1,29) = 2.53, *p* = .116).

We attempted to minimize differences in parents' and children's experiences in the two class types by including the same teacher for all classes: the first author (S.M.), who holds a bachelor's degree in music education and has extensive teaching experience with young children, including in a visual arts-based Reggio Emilia program. While we cannot be certain that the quality of teaching was identical across curricula (S.M. has more teacher training in music than in visual arts), the use of a single teacher should minimize differences in parent-teacher and student-teacher interpersonal relationships across the two class types, two important aspects of participants' training experiences (we test for such differences below).

#### Music curriculum

The music curriculum was modeled after the Eastman Community Music School's Early Childhood Music Program in Rochester, NY (curricular information is available in [34]–[36]). This program adheres to the National Association for the Education of Young Children (NAEYC)'s definitions of developmentally appropriate practice [37], and is consistent with typical music enrichment curricula for preschool children, such as Kindermusik [38], Music Together [39], Orff-Schulwerk [40], and others (for review, see [1], [30]). Additionally, the program emphasizes several content standards outlined in the National Standards for Arts Education: that children should sing a varied repertoire of music alone and with others; listen to, analyze, and describe a varied repertoire of music; and evaluate music and music performances [41]. Further information about the program upon which our music curriculum was based, including teacher interviews and classroom videos, is available in [42].

A typical lesson consisted of welcome activities with both songs and recorded music; gross motor movement activities with group song (e.g., walking, running, rocking, swaying); free-form dancing with recorded music; instrument play with shakers and/or sticks, with songs and recorded music; rhymes and songs with fine motor activities (i.e., “fingerplays”); and a closing activity with a lullaby and goodbye song. Songs were also used to facilitate transitions between class activities. Lesson plans for the program are available on request.

Activities were designed to foster musical play between parent and child, and tended to be short and repeated both within and across sessions to encourage learning of musical repertoire. New songs were introduced on a weekly basis and reinforced in following classes, supporting a sequential six-week curriculum, and handouts with music notation and lyrics were provided to parents on a biweekly basis to reinforce learning of the repertoire. While most children participated in all group activities, this was not required; some children took breaks by playing privately with their parents or exploring the room. This relatively free-form style of instruction is intentional, to immerse the child in a musical environment while parents gain a detailed understanding of the repertoire presented, so that they may incorporate the activities into the home environment [43]–[44]. Indeed, many parents contacted the teacher outside of class with questions and comments about the content of the music classes; see Discussion. Visual arts media were never included in the music class.

#### Visual arts curriculum

The visual arts curriculum paralleled the music curriculum's emphasis on parent-child interaction by encouraging artistic play through visual art media. The teacher modeled a suggested art activity, gave instructions, and participated in projects with parents and children, but did not seek to directly improve their art as a formal teacher might. This informal style is typical of widely employed art curricula in preschool classrooms (e.g., Reggio Emilia programs, see [45]). Like the music curriculum, the visual arts curriculum emphasized several content standards outlined in the National Standards for Arts Education: children should learn to understand and apply visual arts media, techniques, and processes; use knowledge of visual arts structures and functions; choose and evaluate a range of subject matters, symbols, and ideas; and reflect upon and assess the characteristics and merits of their work and the work of others [41].

Due to the more independent nature of most art projects, the teacher played a less active role in moment-to-moment coordination of activities in the visual arts class than in the music class. A rich artistic environment was provided, including many visual arts/constructive materials, allowing children to create both 2-D and 3-D structures (e.g., construction paper, card stock, markers, crayons, colored pencils, paints, felt, feathers, pom-poms, wooden sticks and spoons, stickers, chalkboards, clay, LEGOs, wooden and cardboard building blocks). A typical lesson consisted of a suggested group art project (e.g., masks, murals, clay sculptures) and parent-child play.

As in the music class, most children participated in the suggested activities, but this was not required; some children chose to engage in other related activities instead. Some suggested projects garnered participation from most children (e.g., a group mask-making project), while others garnered less (e.g., a chalk-drawing project). A small number of children (1–2 per class) rarely participated in the suggested activity, but instead chose the same free play activities each week (e.g., building towers with wooden blocks). Children were encouraged to take completed art projects home. As in the music class, parents contacted the teacher with questions and to recommend their children's favorite media (usually made available in subsequent classes). Music was never included in the visual arts class.

#### Posttest

All participants returned for posttests within a week of the final day of classes (on either Feb. 17, *n* = 3; or Feb. 19, *n* = 26; 100% attendance). Treatment type was counterbalanced across morning versus afternoon posttest times. All experimenters administering posttests were blind to treatment condition (music or visual arts). Four assessments were given during the posttest: map use/navigation, 2-D visual form analysis, numerical quantity discrimination, and receptive vocabulary. In addition, we conducted a brief child interview to assess experience in the classes. No further assessments were made of the children. The order of children's first test was counterbalanced across participants and subsequent tests were administered in a pseudorandom order as testing rooms and experimenters became available.

#### Map Use/Navigation test

This test measured children's ability to use purely geometric 2-D maps (devoid of landmarks) to navigate in a 3-D environment. The method followed that of Exp. 2 in [46], with two minor changes: (1) to reduce the length of the test, two training trials (tested first, with corrective feedback) and nine test trials (with neutral feedback) were given; (2) the maps and arrays were roughly half the size of the previous version of the test; the scaling relation (1∶10) between the map and array was preserved.

On each trial, children sat at a table facing away from an array of buckets on the floor and viewed a simple overhead map of the array, with buckets depicted as circles. Training arrays consisted of two differently colored buckets, depicted as differently colored circles on the map. Test arrays consisted of three identical buckets arranged in a line, right triangle, or isosceles triangle and depicted as three identical gray circles. Children viewed a novel map in each trial. The experimenter pointed to one of the circles on the map and instructed the child to place a toy in that location in the room. Maps were presented in one of four orientations: 0°, 90°, 180°, or 270° rotation relative to the array. The orders of target locations, array types, and map orientations were counterbalanced across participants.

#### Visual Form Analysis test

This test measured sensitivity to geometric properties in visual forms through a deviant detection paradigm, and was identical to Exp. 1 in [47] with one minor change: one display was changed from a test trial to a training trial, yielding 3 training trials (tested first, with corrective feedback) and 30 test trials (with neutral feedback). On each trial, children were presented with a display of six different images on a computer screen. Five images illustrated a given geometric property (e.g., parallel lines), while one image differed on that property (e.g., perpendicular lines). Children were instructed to point to the item that looked different. Children received four trials testing sensitivity to topology, five to angle, eight to distance, five to sense relations, and eight to straight lines/parallelism. The order of the test trials was randomized and the location of the correct response was counterbalanced within-subjects.

#### Numerical Discrimination test

This test measured non-symbolic numerical discrimination ability using the method of [26]. On each trial, two arrays of dots appeared side-by-side on the screen of a laptop computer, in the context of a game in which the child's goal was to identify which of two characters had more dots (Big Bird or Grover). Arrays of dots were presented in four ratios: 1∶2, 2∶3, 3∶4, and 4∶5, and ranged in numerosity from 4 to 15 dots per side. The test included 6 training trials and 60 test trials. The program provided feedback for each trial (high-pitched beep for correct or low-pitched beep for incorrect), and the experimenter gave occasional neutral feedback to maintain motivation. The location of the more numerous array (and thus its color and character association), as well as whether the more numerous set or less numerous set had greater surface area, was counterbalanced across trials. Children entered their responses by pushing a button to indicate which character had more dots.

#### Peabody Picture Vocabulary Test

The PPVT was used to measure receptive vocabulary, and is an individually administered, untimed, norms-referenced test in two parallel forms (A and B), used as an achievement test of receptive (hearing) vocabulary attainment for Standard English, and/or as a screening test of verbal ability [16]. The PPVT-IIIa was administered at pretest (see above) and the PPVT-IIIb at posttest. Each test item consists of four black-and-white illustrations arranged on a page; the child is asked to select the picture that best represents the word spoken by the experimenter. The test was administered as specified in [16].

#### Child interview

To measure children's perceptions of their classes, we interviewed children with a 5-point pictorial Likert-type scale using faces with a gradient of emotional expression, from frown (“very sad”) to smile (“very happy”). The experimenter trained children to use the scale via practice questions with standard answers and corrective feedback if necessary (i.e., “If I gave you a really cool sticker, how would you feel?”). Three test questions followed: (1) “Remember the other kids in the class? How do you feel about the other kids in the class?” (2) “Remember the teacher, Sam? How do you feel about Sam?” and (3) “If the class happened again, and you came back to class here again, how would you feel?” Questions were asked in the above fixed order.

### Results

Participants were randomly assigned to the music or visual arts group, such that there were no significant differences between children's PPVT-IIIa score, family income, parents' AMMA scores, parents' level of education, or parents' number of working hours per week. Groups were also matched for age, although after the attrition of three participants, the age of children in the music group was slightly higher (*t*(27) = 2.05, *p* = .052; see Table 2). As expected, children's performance on the parallel forms of the PPVT before and after arts training was highly correlated (*r* = .785, *p*<.0001). No significant correlations were found between the other tests.

**Figure 1:**
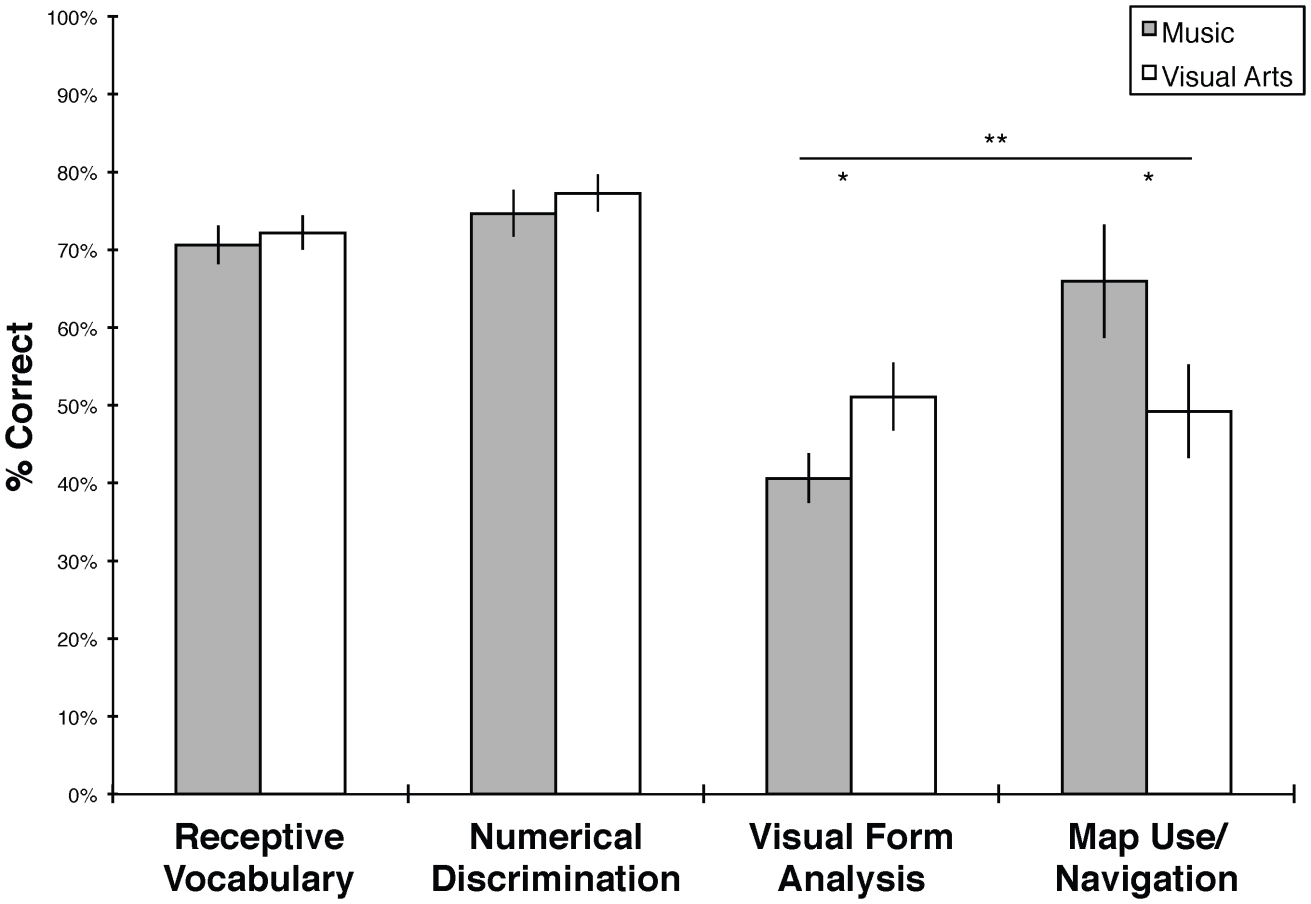
Mean test performance by group in Experiment 1. Scores are reported as total percent correct. PPVT-IIIb scores are standardized by age and calculated as percent of the highest possible standard score. Error bars denote standard errors of the mean. **p<.01; *p<.05, one-tailed.

The main findings are presented in Figure 1 and descriptive statistics are presented in Table 3. First, we conducted a 4×2 multivariate analysis of variance (MANOVA) with Visual Form Analysis, Map Use/Navigation, Numerical Discrimination, and Receptive Vocabulary tests as dependent measures, and Group (music or visual arts) as a between-subjects factor. Results showed no significant effects (Wilks' λ = .742, *F*(4,24) = 2.09, *p* = .113).

**Table 3:** Mean posttest performance in Experiments 1 and 2.

	Experiment 1		Experiment 2	
Assessment	Music	Visual Arts	Music	Control
PPVT-IIIb (age-standardized)	113 (15.4)	116 (13.3)	116 (14.4)	120 (14.0)
Numerical Discrimination (% correct)	74.7 (11.7)	77.3 (8.98)	80.9 (11.6)	81.1 (10.0)
Visual Form Analysis (% correct)	40.6 (12.4)	51.1 (16.4)	44.6 (16.8)	44.2 (14.5)
Map Use/Navigation (% correct)	65.9 (28.3)	49.2 (22.5)	62.3 (25.2)	63.1 (23.1)

*Note*. Standard deviations are in parentheses. As in Table 2, PPVT scores are standardized by age.

To determine whether arts training had a specific effect on spatial reasoning, as opposed to numerical or verbal reasoning, we conducted a follow-up analysis that focused on children's performance on the two assessments of spatial cognition. We performed a 2×2 repeated-measures ANOVA with Spatial Assessment (Visual Form Analysis or Map Use/Navigation test, standardized as z-scores) as a repeated measure, and Group (music or visual arts) as a between-subjects factor. Mauchly's test indicated that the assumption of sphericity was not violated. Results showed a significant interaction between Spatial Assessment and Group (*F*(1,27) = 9.009, *p* = .009), with no other significant effects. On the Map Use/Navigation test, children in the music group performed significantly better than those in the visual arts group (*t*(27) = 1.75, *p* = .031; one-tailed, *d* = 0.65). In addition, children in the visual arts group performed significantly better than those in the music group on the Visual Form Analysis test (*t*(27) = −1.95, *p* = .045, one-tailed; *d* = 0.72). In contrast, the music and visual arts groups showed no significant differences in performance on the PPVT-IIIb or the Numerical Discrimination test (PPVT-IIIb: *t*(27) = −.466, *p* = .645; Numerical Discrimination: *t*(27) = −.668, *p* = .510). Sensitivity analyses revealed that these results were not attributable to the presence of influential observations.

The child interview revealed no significant differences between groups, as measured by Wilcoxon rank-sum tests, in terms of level of positive feeling toward the other children in the class (*z* = −1.06, *p* = .289) or of the teacher of the class (*z* = 1.60, *p* = .111). However, children in the visual arts group reported that they would feel happier to return and participate in the same class again than did children in the music group (*z* = 2.81, *p* = .005). Despite the teacher's more extensive training in music, therefore, the visual arts classes generated at least as much student interest and engagement as did the music classes.

### Discussion

Experiment 1 provides suggestive evidence for two effects of preschool arts instruction on young children's spatial cognitive abilities. Four-year-old children who completed six weeks of a typical developmentally appropriate preschool music class showed greater ability to use an abstract geometrical map to navigate in a 3-D layout relative to children in a similar class focused on visual arts. In addition, children who participated in the visual arts class showed greater ability to analyze the geometrical properties of 2-D visual forms, relative to children in the music class.

However, we present these findings with two caveats. First, both differences in performance were statistically weak. Had these findings not been predicted based on past research, they would not have survived correction for multiple comparisons. Second, all children in Experiment 1 participated in either music or visual arts classes; thus, these data do not provide a comparison to children's performance in a no-treatment control group, complicating attributions of causality. To remedy these problems, we conducted a second randomized trial substituting a no-treatment control for the visual arts group, and testing a larger sample of participants.

## Experiment 2

### Methods

#### Participants

Recruitment methods were identical to Experiment 1. Approximately 50 families responded, of which 46 were invited to participate in the full study on the same basis as Experiment 1. One child in the music group discontinued participation after the first day of classes, for a 2.2% rate of attrition (after attrition: music group: *n = *23, 10 female; control group: *n* = 22, 11 female).

#### Pretest and Subject Assignment

Figure S2 describes the chronology of participation in Experiment 2. Pretests were identical to Experiment 1 and took place between December 5, 2011 and January 14, 2012. Due to a more in-depth phone interview before the pretest session, no children were excluded from the study following the pretest. As in Experiment 1, participants were randomly sorted into two groups (music or control), such that extant differences between groups were minimized. In Experiment 2, we expanded our MATLAB script to balance group characteristics of age, gender, family income, ethnicity, child PPVT-IIIa score, parent AMMA score, bilingualism, number of siblings, parent education, number of parent work hours, and gender of primary parent (i.e., the parent who would attend class with their child).

#### Training

The music group was provided with identical training to Experiment 1. Three music classes were conducted, each with six weekly 45-minute sessions held on Saturday mornings (February 11—March 17, 2012). The same curriculum, teacher, class size (seven or eight children per class), and classroom were used as in Experiment 1. Attendance was high in the music classes (89%; 15 absences and 123 attendances), with no significant difference from the rate of attendance to either the music or visual arts classes in Experiment 1 (Music: χ^2^(1,45) = .597, *p* = .440; Visual Arts: χ^2^(1,45) = 1.01, *p* = .316). The control group did not participate in any training prior to data collection; to motivate participation in the study, participants in the control group were provided with music classes in the six weeks following the posttest. To help control for children's familiarity with our lab between groups, an informal “meet and greet” was provided for the families in the control group, where parents met staff and graduate students who were not involved with the study (thus, familiarizing parents with the lab while maintaining experimenter blindness) and children played together in a playroom. Nine families from the control group attended this event, for a 41% rate of attendance.

#### Posttest

All participants returned for posttests within eight days of the final day of classes for the treatment group (on either March 22, *n* = 4; March 24, *n* = 22; or March 25, 2012, *n* = 19; 100% attendance). Treatment and control groups were counterbalanced across morning versus afternoon posttest times. All experimenters administering posttests were blind to condition (treatment or control). Four assessments, identical to Experiment 1, were given (map use/navigation, 2-D visual form analysis, numerical quantity discrimination, and receptive vocabulary). No further assessments were made of the children.

The order of children's first test was counterbalanced across participants and subsequent tests were administered in a pseudorandom order as testing rooms and experimenters became available. The same testing rooms were used for each task as in Experiment 1, and two of the four assessments (visual form analysis and receptive vocabulary) were given by the same experimenters as in Experiment 1.

### Results

Participants were randomly assigned to the music or control group such that there were no significant differences between groups on any group characteristics (see Table 2). As in Experiment 1, children's performance on parallel forms of the PPVT-III was highly correlated (*r* = .682, *p*<.0001). Performance on the Visual Form Analysis and Numerical Discrimination tests was also correlated (*r* = .611, p<.0001); no other significant correlations between assessments were found.

**Figure 2:**
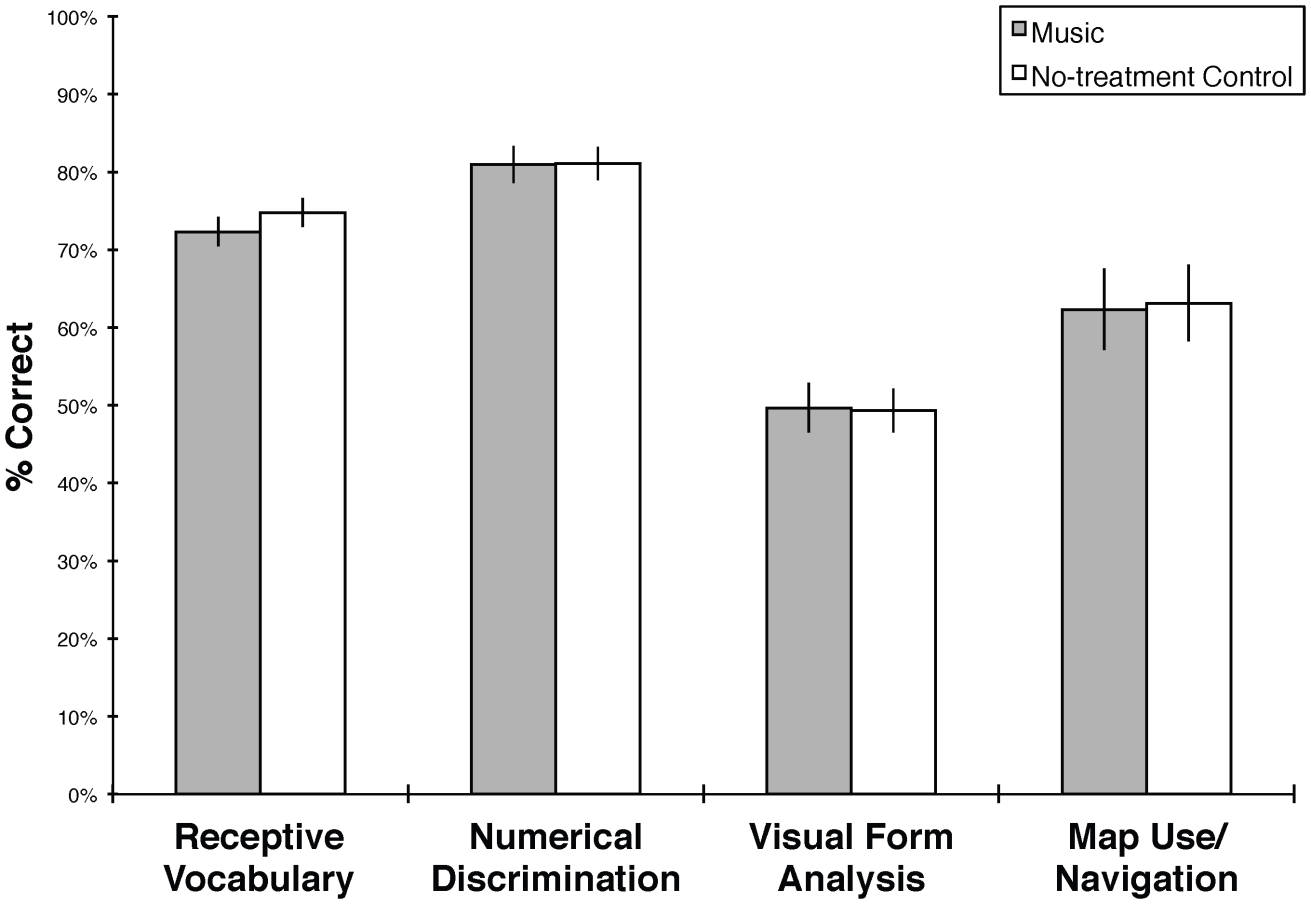
Mean test performance by group in Experiment 2. Scores are reported as total percent correct. PPVT-IIIb scores are standardized by age and calculated as percent of the highest possible standard score. Error bars denote standard errors of the mean.

The main findings are presented in Figure 2 and descriptive statistics are presented in Table 3. We conducted the same analyses as in Experiment 1, beginning with a 4×2 MANOVA with Visual Form Analysis, Map Use/Navigation, Numerical Discrimination, and Receptive Vocabulary tests as dependent measures, and Group (music or visual arts) as a between-subjects factor. Results showed no significant effects (Wilks' λ = .980, *F*(4,40) = 0.20, *p* = .934). We then conducted a 2×2 repeated-measures ANOVA with Spatial Assessment (Visual Form Analysis or Map Use/Navigation test, standardized as z-scores) as a repeated measure, and Group (music or visual arts) as a between-subjects factor. In contrast to Experiment 1, the results showed no significant interaction between Spatial Assessment and Group (*F*(1,43) = .02, *p* = .887) and follow-up *t*-tests showed no significant differences between groups on any assessment (PPVT-IIIb: *t*(43) = −.921, *p* = .362; Numerical Discrimination: *t*(43) = −.037, *p* = .971; Map Use/Navigation: *t*(43) = −.113, *p* = .911; Visual Form Analysis *t*(43) = .084, *p* = .933). Sensitivity analyses revealed that these results were not attributable to the presence of influential observations.

### Combined Analyses of Experiments 1 and 2

No significant differences in task performance or in any demographic characteristics were found between the music groups from Experiments 1 and 2 (*p*s>.1). Thus, we conducted further analyses with a combined music group (*n* = 38), the visual arts group (*n* = 14), and the no-treatment control group (*n* = 22). We conducted a 4×3 MANOVA with Visual Form Analysis, Map Use/Navigation, Numerical Discrimination, and Receptive Vocabulary tests as dependent measures and Group (combined music, visual arts, or control) as a between-subjects factor. Results showed no significant effects (Wilks' λ = .851, *F*(8,136) = 1.43, *p* = .190). The lack of significant effects did not appear to be due to floor or ceiling effects: children performed significantly above chance and below ceiling on all tests (*p*s<.00001).

Post-hoc analyses confirmed the lack of significant differences between the combined music group and the control group, (PPVT-IIIb: *t*(58) = −1.29, *p* = .204; Numerical Discrimination: *t*(58) = −.863, *p* = .392; Map Use/Navigation: *t*(58) = .091, *p* = .928; Visual Form Analysis: *t*(58) = −.846, *p* = .401), between the combined music group and the visual arts group, (PPVT-IIIb: *t*(50) = −.194, *p* = .847; Numerical Discrimination: *t*(50) = .344, *p* = .732; Map Use/Navigation: *t*(50) = 1.84, *p* = .072; Visual Form Analysis: *t*(50) = −1.05, *p* = .300), or between the visual arts group and control group (PPVT-IIIb: *t*(34) = .870, *p* = .391; Numerical Discrimination: *t*(34) = 1.15, *p* = .257; Map Use/Navigation: *t*(34) = 1.78, *p* = .084; Visual Form Analysis: *t*(34) = −.357, *p* = .723).

**Figure 3:**
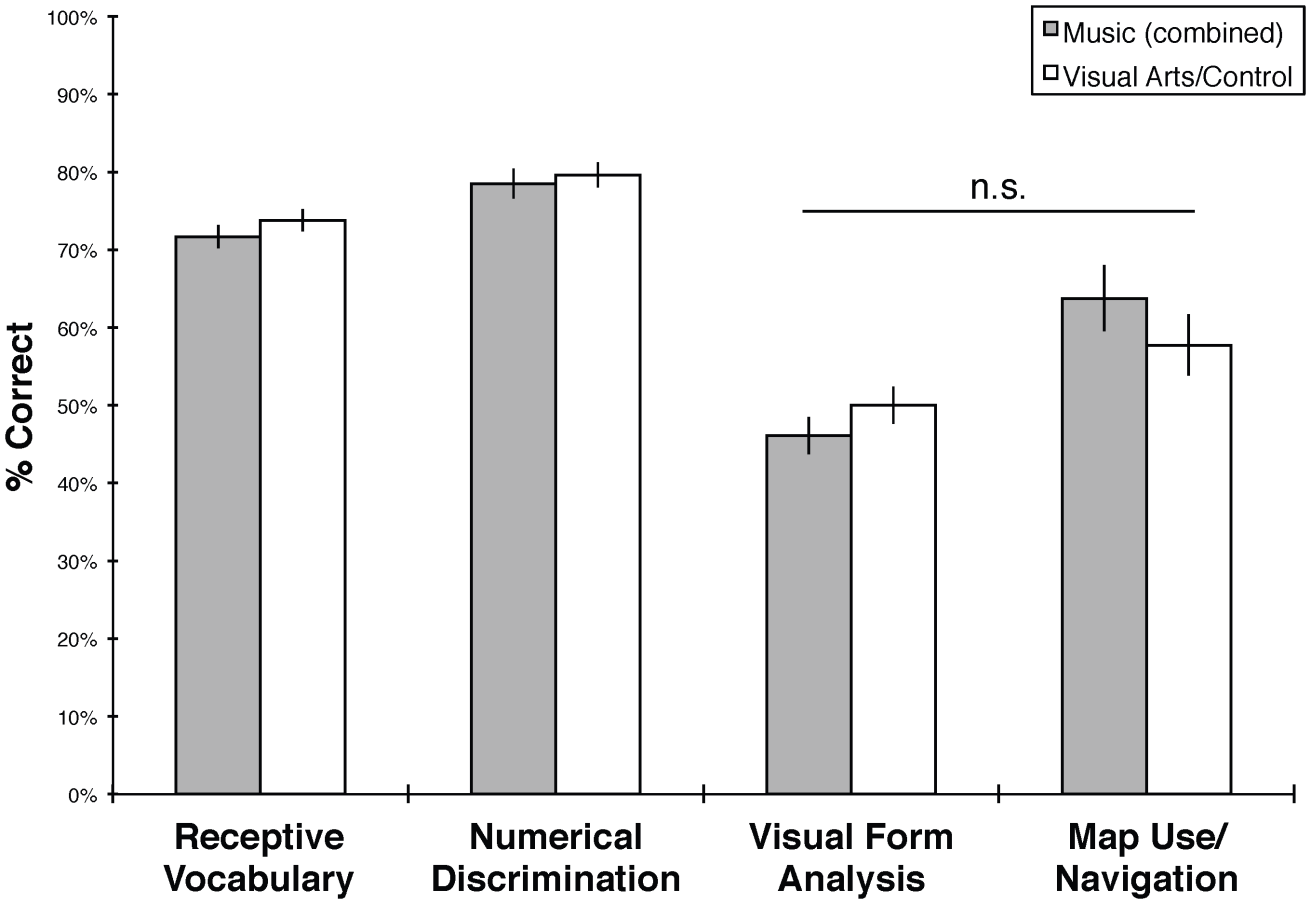
Overall results, combining music and comparison/control groups. Scores are reported as total percent correct. PPVT-IIIb scores are standardized by age and calculated as percent of the highest possible standard score. Error bars denote standard errors of the mean.

Given comparable performance between the comparison (visual arts) group and no-treatment control (see above), we conducted further analyses that compared children in the combined music training group (*n* = 38) to a combined control group (*n* = 36). This practice is comparable to the main analysis in [7], which compared performance of a combined music group with a combined comparison/control group. We performed a 2×2 repeated-measures ANOVA with Spatial Assessment (Visual Form Analysis or Map Use/Navigation test, standardized as z-scores) as a repeated measure, and Group (combined music or combined visual arts/control) as a between-subjects factor. Mauchly's test indicated that the assumption of sphericity was not violated. Results showed no significant interaction between Assessment and Group (*F*(1,72) = 2.27, *p* = .137). Post-hoc analyses revealed no significant differences in performance on any test (PPVT-IIIb: *t*(72) = 1.02, *p* = .311; Numerical Discrimination: *t*(72) = .443, *p* = .659; Map Use/Navigation: *t*(72) = −1.04, *p* = .303; Visual Form Analysis: *t*(72) = 1.15, *p* = .252). These results appear in Figure 3.

## General Discussion

The current report provides no consistent evidence for cognitive transfer from music training: preschool music classes did not cause detectable skill increases in the cognitive domains of spatial, linguistic, or numerical reasoning. We assessed transfer effects of music education by measuring cognitive skills in specific domains in preschoolers, after completing an ecologically representative program of parent-child music enrichment. We conducted two randomized trials, which together included both a comparison group with alternate (visual arts) training, and a no-training control. While the results from our first trial appeared to show effects of arts instruction on two spatial abilities, consistent with past correlational research [28], our second, more powerful follow-up trial failed to support this finding. Together, these findings suggest that preschool music education may not increase the spatial, linguistic or numerical skills measured herein, and underscore the importance of replication and correctly implemented control groups in studies assessing the cognitive benefits of educational programs.

In contrast to previous research, we tested children on specific areas of cognition as opposed to general intelligence. Our approach did not simply tap domain-general abilities in four separate contexts, as evidenced by our analysis of the relationships between our outcome measures: with only one exception, no two outcome measures were correlated. Our design therefore had the potential to uncover transfer effects from music education in specific areas of cognition, in contrast to previous research. However, despite this novel method and the examination of four different domains, we found no consistent evidence for cognitive transfer from music training.

Of course, these negative findings do not imply that preschool arts instruction does not engender nonmusical cognitive benefits to preschool children. One concern is that we might have observed cognitive benefits of music classes had the classes had continued for a longer time. While the total classroom time in each of our experiments was less than those of previous randomized trials, in previous studies longer music training duration did not necessarily yield larger transfer effects, even after similar training curricula (see Table 1): [5], [7] employed similar means of music training (piano lessons), but only [7] reported a positive overall transfer effect, despite the fact that training duration was twice as long in [5]. Likewise, [6], [8] both employed classroom-based group music classes, the training in [8] was approximately 50% longer, but neither study found a positive overall transfer effect. The lack of relationship between training duration and effect size is further evidenced by repeated-measures analyses in the only multi-year RCT on the topic: a positive transfer effect was found after two years of piano lessons, but this effect disappeared after an additional year of training [5]. We acknowledge, however, that our training duration is substantially shorter than previous RCTs, and note that several possibilities exist that may undermine our ability to detect a transfer effect. For instance, it is possible that the relationship between training duration and transfer effect size follows a step function, such that a minimum duration of training is necessary to elicit transfer effects; this explanation might account for our negative results.

A second concern is that we might have observed transfer effects had our music curriculum involved more intense music instruction. Here we note first that our training curricula were consistent with two previous RCTs: [6], [8] both used classroom-based group music instruction in lieu of instrumental music lessons. Our studies differ in one key respect, however, as we included parents in each class and encouraged parents to incorporate music into the daily lives of their children at home. In this respect we were successful, as evidenced by regular contact between, parents and the teacher in the days between class meetings, where parents asked the teacher for information and assistance with music from the class repertoire. This is a pattern typical of parent-child music classes [1], [30], [43]–[44] and is consistent with the design of the program upon which our curriculum was based [34]–[36]. Thus, we assess cognitive benefits of an ecologically representative early childhood music enrichment program of the type sanctioned by music educators worldwide (see Method).

We acknowledge, however, that our training intensity differs from previous work employing more formal music instruction. It is possible that intense training of the type traditionally reserved for older children might elicit cognitive benefits in preschool children; indeed, the only RCT reporting a positive overall effect of music training on IQ included formal piano and voice instruction in a conservatory setting [7]. However, it is also possible that intense training at such an early age could have *negative* effects on children's cognitive skills, as they might find such intensity aversive, leading to lower performance on cognitive assessments. The relation between training intensity and cognitive transfer thus remains unclear.

A third concern stems from our choice of outcome measures and the timing with which we gave those measures. The lack of consistent positive effects in our studies might be due to our choice to use tests of specific cognitive abilities instead of a general IQ measure (although the PPVT correlates highly with measures of IQ: for instance, correlations of WISC-III [10] index scores with the PPVT-III range from.82 to.92 [16], this IQ measure was used in two of five previous RCTs; see Table 1). We chose to measure receptive vocabulary, numerical cognition, and two forms of spatial cognition because these abilities are highly sensitive to other manipulations and factors affecting young children's cognitive performance [16], [24]–[26]. In addition, high school students' participation in music and dance training is associated with higher performance on a navigation task, while the duration of visual arts training predicts performance on a visual form analysis task [28].

A consequence of this decision, however, is that only the PPVT could be administered at both pre- and posttest, with the remaining three tests administered only at posttest. Previous RCTs have tested for cognitive effects of music training via analysis of gain scores [6]–[7] or training type by time interaction [5], [8]–[9], given the inclusion of identical pre- and posttests. Such a study design requires cognitive assessments that produce reliable and valid results even when administered repeatedly. However, our tests of visual form analysis, symbolic navigation, and numerical discrimination include both unique content (e.g., uncommon geometrical figures) and methods (e.g., using a symbolic map to navigate in a room), rendering them highly vulnerable to practice effects. Thus, only the PPVT was administered at both pre- and posttest. This may have decreased the sensitivity of our analyses: it is possible, for instance, that a population effect of music training on symbolic navigation skill exists, but that by random chance the control and visual arts groups had stronger spatial reasoning abilities than the music group at the outset of the study, and thus scored comparably to the music-trained group at posttest. In this scenario, the training and comparison/control groups would have scored differently on a pretest, had one been administered. Given the random assignment to groups and their high degree of similarity on a variety of dimensions (see Table 2), this outcome is unlikely, but we cannot rule it out.

A fourth concern is the lack of an assessment of the *direct* effects of our music curriculum, as a manipulation check. Because the stated goal of our classes was not to improve children's specific musical skills (e.g., improved perception of rhythmic or melodic patterns), but rather to increase the quality and frequency of parent-child musical play, it would be desirable to measure whether such interactions were indeed enhanced by the music classes. We have indirect evidence that our manipulation was successful in this regard: at least 60% of parents participating in the music classes contacted the teacher with questions and comments about course content during the interval between the weekly classes, usually via email. These communications typically consisted of reports of children's favorite song repertoire and requests that the songs be repeated in class, requests for music notation or lyrics to help repeat activities accurately at home, or general comments that indicated participation in musical play outside of class. Other parents made similar communications in person or by phone. This indirect evidence, consistent with programs with similar curricula [1], [34]–[35], suggests that the music classes had their intended effect.

Lastly, we note the possibility of “sleeper effects”: there may be effects of brief musical experiences that do not emerge immediately following music training. For example, parents who participate in a short music enrichment program may be more motivated to seek out musical experiences for their children over a number of years, and these experiences could provide a variety of benefits that have not yet been identified in the literature. As a second example, children who participate in a short music enrichment program could develop a more positive attitude toward group learning situations, and this attitude may foster their later learning in school settings. To our knowledge, no study has yet investigated the existence or extent of such effects; this area should be addressed in future RCTs.

When taken together with existing literature, the current experiments are the sixth and seventh attempt to study the cognitive effects of music training via RCTs. We add a negative finding to the small body of randomized trials on the subject, complicating an already unclear pattern of results, but helping to resolve a potential publication bias in this literature [14]–[15]. Further RCTs are necessary to determine the existence and extent of extrinsic cognitive benefits of music education in childhood, as well as the *musical* benefits of musical experiences. Regardless of any potential transfer effects, we echo the view of Winner and Hetland [48] that the primary benefit of music education for parents and children is self-evident: to improve the musical skills and repertoire of parents and children along with their appreciation and enjoyment of musical activities. Whether or not future studies uncover reliable relations between music education and extra-musical aspects of cognitive development, instruction in the arts likely will thrive for its intrinsic value.

## Supporting Information

Figure S1 **Flowchart of participants through Experiment 1.**
(TIFF)

Figure S2 **Flowchart of participants through Experiment 2.**
(TIFF)
