# Supplementary figures and images for: Two Randomized Trials Provide No Consistent Evidence for Nonmusical Cognitive Benefits of Brief Preschool Music Enrichment
Source: PLoS One. 2013 Dec 11;8(12):e82007. doi: 10.1371/journal.pone.0082007 (PMC3859544; doi:10.1371/journal.pone.0082007)

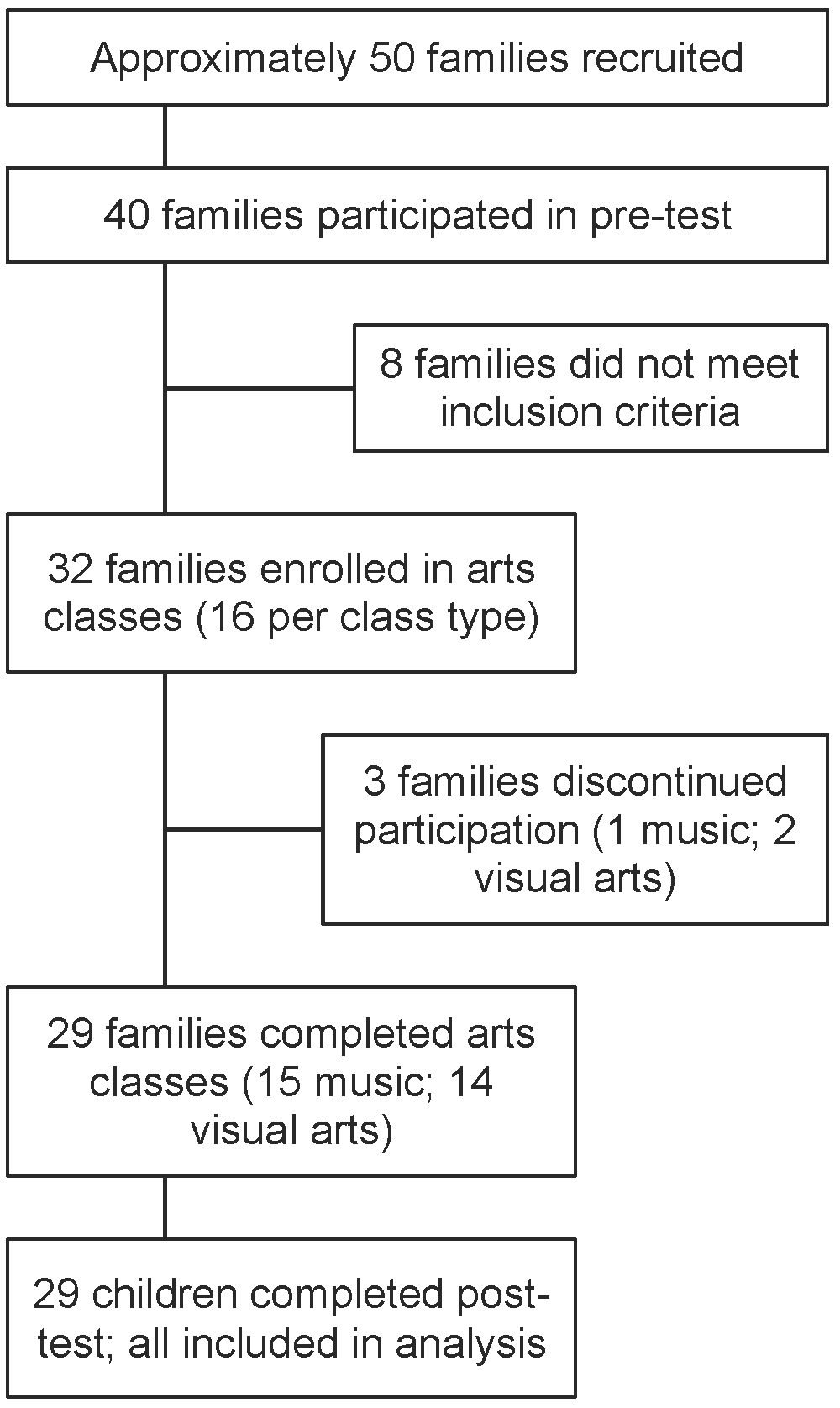

Supplement: Figure S1 — Flowchart of participants through Experiment 1. (TIFF) [file pone.0082007.s001.tiff]

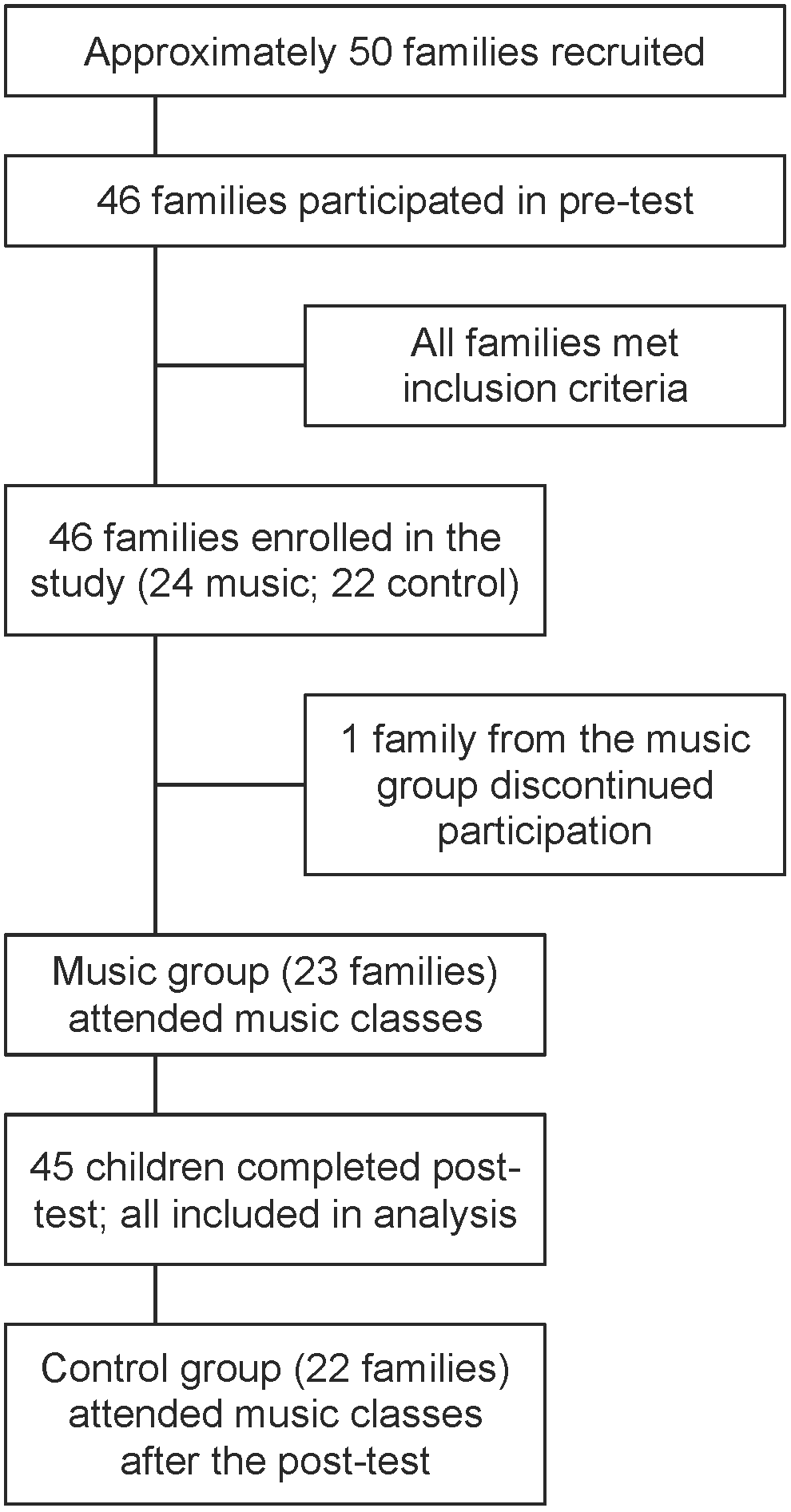

Supplement: Figure S2 — Flowchart of participants through Experiment 2. (TIFF) [file pone.0082007.s002.tiff]
